# A study protocol of the adaptation and evaluation by means of a cluster-RCT of an integrated workplace health promotion program based on a European good practice

**DOI:** 10.1186/s12889-022-13352-0

**Published:** 2022-05-21

**Authors:** Denise J. M. Smit, Sandra H. van Oostrom, Josephine A. Engels, Allard J. van der Beek, Karin I. Proper

**Affiliations:** 1 Center for Nutrition, Prevention and Health Services, National Institute for Public Health and the Environment, Bilthoven, 3721 MA The Netherlands; 2 Department of Public and Occupational Health, Amsterdam UMC, Vrije Universiteit Amsterdam, Amsterdam Public Health Research Institute, Amsterdam, 1081 BT The Netherlands; 3 Occupation & Health Research Group, HAN University of Applied Sciences, Nijmegen, 6525 EN the Netherlands

**Keywords:** Workplace health promotion, Integrated approach, Map of adaptation process, Protocol, Cluster randomized controlled trial, Effect evaluation, Process evaluation

## Abstract

**Background:**

An integrated workplace health promotion program (WHPP) which targets multiple lifestyle factors at different levels (individual and organizational) is potentially more effective than a single component WHPP. The aim of this study is to describe the protocol of a study to tailor a European good practice of such an integral approach to the Dutch context and to evaluate its effectiveness and implementation.

**Methods:**

This study consists of two components. First, the five steps of the Map of Adaptation Process (MAP) will be followed to tailor the Lombardy WHP to the Dutch context. Both the employers and employees will be actively involved in this process. Second, the effectiveness of the integrated Dutch WHPP will be evaluated in a clustered randomized controlled trial (C-RCT) with measurements at baseline, 6 months and 12 months. Clusters will be composed based on working locations or units - dependent on the organization’s structure and randomization within each organization takes place after baseline measurements. Primary outcome will be a combined lifestyle score. Secondary outcomes will be the separate lifestyle behaviors targeted, stress, work-life balance, need for recovery, general health, and well-being. Simultaneously, a process evaluation will be conducted. The study population will consist of employees from multiple organizations in different industry sectors. Organizations in the intervention condition will receive the integrated Dutch WHPP during 12 months, consisting of an implementation plan and a catalogue with activities for multiple lifestyle themes on various domains: 1) screening and support; 2) information and education; 3) adjustments in the social, digital or physical environment; and 4) policy.

**Discussion:**

The MAP approach provides an appropriate framework to systematically adapt an existing WHPP to the Dutch context, involving both employers and employees and retaining the core elements, i.e. the catalogue with evidence-based activities on multiple lifestyle themes and domains enabling an integrated approach. The following process and effect evaluation will contribute to further insight in the actual implementation and effectiveness of the integrated WHP approach.

**Trial registration:**

NTR (trialregister.nl), NL9526. Registered on 3 June 2021.

**Supplementary Information:**

The online version contains supplementary material available at 10.1186/s12889-022-13352-0.

## Background

Non-communicable diseases (NCDs) are the leading cause of death worldwide [1, 2]. Unhealthy lifestyle behaviors are well-known modifiable risk factors of NCDs. Therefore, promotion of a healthy lifestyle is of importance [3, 4]. The workplace is seen as an appropriate setting to promote health including the improvement of a healthy lifestyle [5, 6]. Workplace health promotion programs (WHPPs) can be effective in improving the lifestyle behaviors targeted [7–10]. For instance, a review of reviews by Proper et al. concluded that WHPPs have a positive effect on both body weight-related outcomes and the prevention of mental and musculoskeletal problems [7]. However, it should be acknowledged that in some of these systematic reviews, evidence was limited to moderate [8–10]. Individual participant data meta analyses from Robroek et al. and Coenen et al. even showed that overall there was no statistically significant effect of WHPPs on BMI, physical activity, alcohol consumption, smoking and diet, with the exception of fruit intake [11, 12]. Most interventions included in these reviews focused on the individual or environmental level only. The abovementioned findings indicate that there is a need for new directions in the design of WHPPs [11, 13].

A greater impact on lifestyle and health can be expected from an integrated approach, which targets the individual level as well as the organizational level [14]. Earlier studies have indeed shown greater effects of WHPPs that focus on an environmental component in addition to individually based components on the targeted lifestyle behaviors [8, 15, 16]. Nevertheless, these interventions often include only minimal environmental changes. More extensive environmental changes are necessary [13]. A good example of a successful integrated WHPP is the Lombardy WHP Network, which is recognized as a good practice in the occupational setting in the European Joint Action CHRODIS because of its integrated approach and successful implementation [14, 17]. This program has been implemented in Lombardy, Italy, where participating organizations received a catalogue in which activities on both the individual and organizational level for multiple lifestyle themes are included. Employers chose which activities to implement at both the individual and organizational level. A pilot study with a follow-up of 1 year showed significantly positive effects on smoking cessation and fruit and vegetable intake, and favorable changes were apparent for alcohol intake and physical activity [18]. The Lombardy WHP Network was further successful in the implementation and participation of organizations [19]. Development of the program started in 2011 in Bergamo, and in 2013 it expanded on a regional scale. In 2014, 284 workplaces, employing 139,186 persons, were involved [14, 19]. The catalogue with evidence-based activities was continuously updated, which also contributed to the success of the Lombardy WHP Network [19]. The catalogue may also have played a role in the successful implementation, due to the wide range of small and accessible WHP activities provided, an integrated approach that fits the organization can be composed. Such a WHPP is possibly easier to implement when compared to an imposed extensive WHPP. A similar integrated WHPP, based on the Lombardy WHP Network, has been implemented in Andalusia, Spain [20]. Initial results after a nine-month implementation period showed no statistically significant changes yet, but the frequency of sweets consumption within one organization declined with 6.2% (10.8% vs 4.6%) and physical activity in the same organization increased with 12.3% (23.1% vs 35.4%) [21]. Currently there is a lack of such integrated WHPPs and scientific evidence about their effectiveness and implementation [13].

Because of the integrated approach, successful implementation and effects on lifestyle behaviors, our aim was to describe the protocol of a study to tailor the integrated European good practice Lombardy WHP Network to the Dutch context and to evaluate its effectiveness and implementation by means of a cluster randomized controlled trial. This paper describes two components: 1) the protocol of the systematic tailoring of the Lombardy WHP Network to the Dutch context, and 2) the design of the effect and process evaluation.

## Methods/design

**Fig. 1:**
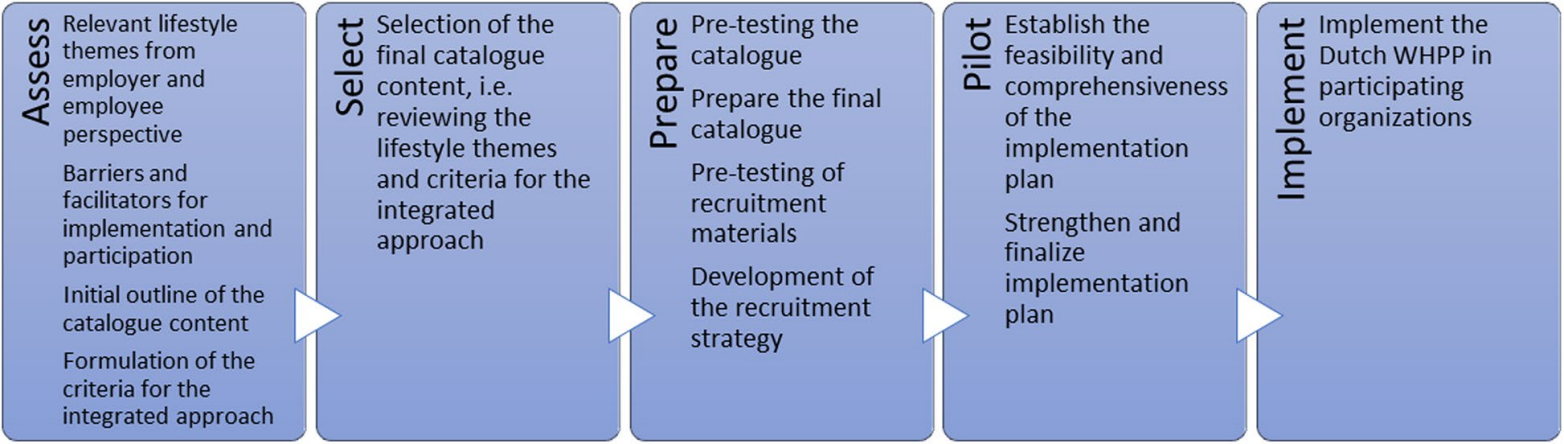
Steps from the map of adaptation process

For the first component of this study, the protocol of the systematic tailoring of the Lombardy WHP Network, the Map of Adaptation Process (MAP) will be followed. The MAP is a stepwise and systematic approach for the adaptation of an evidence-based behavioral approach to new contexts [22]. The MAP allows a bottom-up approach, in which stakeholders, such as the employers and employees, will be involved in the different steps [23]. Hence, the program can be tailored to their needs and preferences. The MAP consists of five steps: 1) assessment of relevant lifestyle themes, potential barriers and facilitators for implementation and participation, potential activities to be included in the catalogue and the formulation of criteria for an integrated WHPP in the Dutch context, 2) selection of the final content for the Dutch context adapted catalogue, 3) preparation of the catalogue for implementation, 4) pilot test of the feasibility and comprehensiveness of the implementation plan, and 5) implementation of the program (Fig. 1).

### Tailoring of the Lombardy WHP network to the Dutch context

#### Step 1. Assess

Based on the Lombardy WHP Network, the Dutch WHPP will consist of a catalogue along with an implementation plan to support successful implementation. For the development of the catalogue, lifestyle themes relevant for both the employers and employees will be established. Also, potential barriers and facilitators for implementation of and participation in WHP activities will be identified, these will be used to develop the implementation plan. The catalogue will consist of effective activities to improve lifestyle, an initial draft for the catalogue content will be comprised. Criteria that organizations must fulfill in order to implement activities according to integrated approach in the Dutch WHPP will be formulated.

To identify the relevant lifestyle themes and the barriers and facilitators for implementation of and participation in WHPPs, focus groups with employers and peer-to-peer interviews with employees will be conducted. Focus group will be carried out with managers, HR professionals and prevention workers, whom in this study represent the employers’ perspective. A variety of organizations with both blue collar and white collar employees will be represented in these focus groups. In addition, peer-to-peer interviews, in which employees interview their co-workers will be conducted. Peer-interviewers will be recruited within different organizations and departments, to ensure they represent various job types and educational levels. Peer-to-peer interviewing is a method derived from citizen science, in which participants actively take part in conducting research [24]. Advantages are an efficient data collection and less socially desirable answers as persons are considered to respond more genuinely to their peers [24–26].

A toolkit with WHP activities, developed in 2020 within the Joint Action CHRODIS PLUS [27], will be used as a starting point for the initial draft of the catalogue content together with results from the focus groups and peer-to-peer interviews. The WHP activities will be tailored to the Dutch context.

**Fig. 2:**
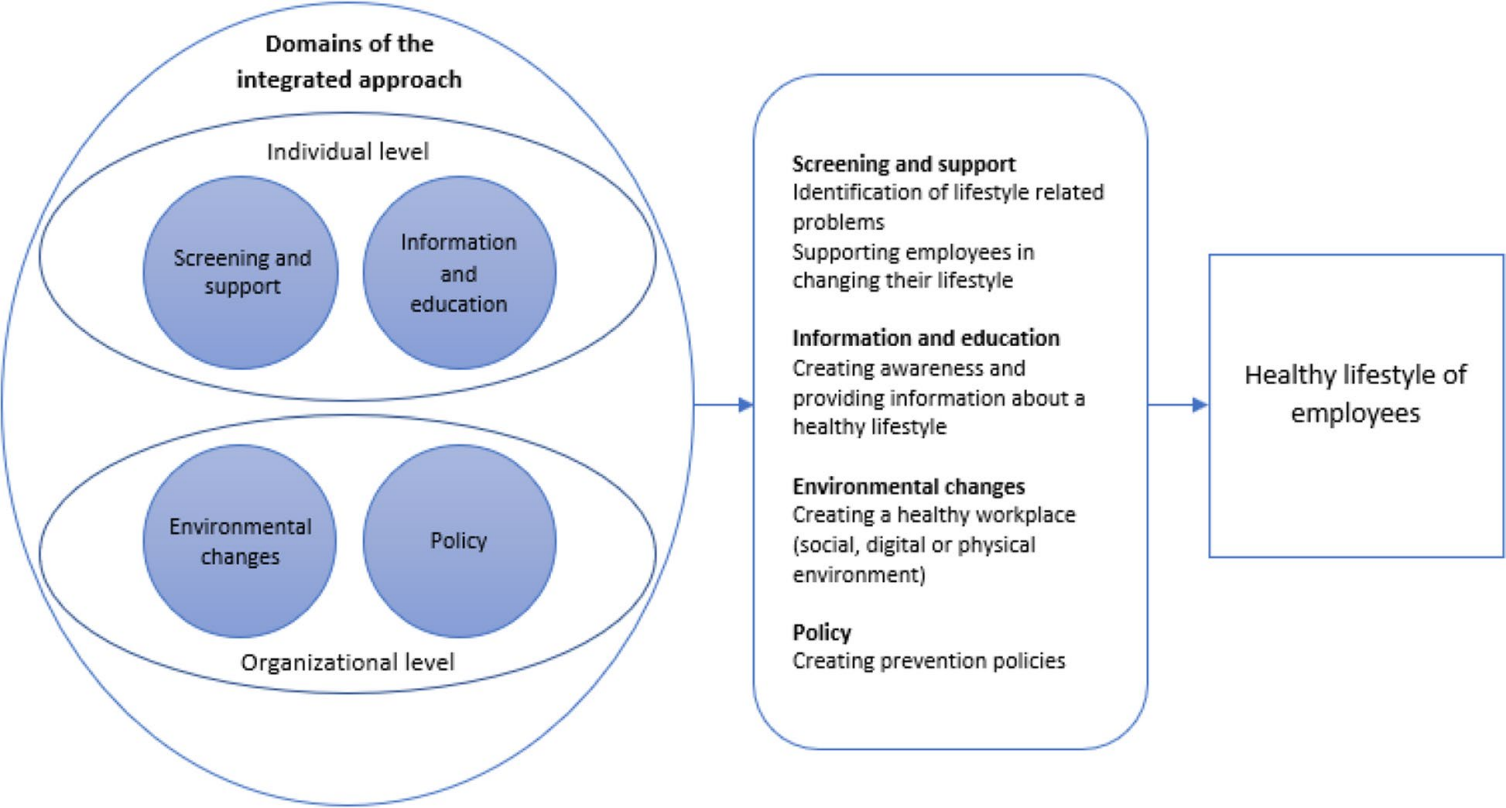
Model of the integrated approach

The criteria of the integrated approach in the Dutch context will be formulated by the researchers based on the definition for an integrated approach of the Lombardy WHP Network and the definition of other Dutch integrated health promotion programs developed by the National Institute of Public Health and the Environment, Center of Healthy Living [28, 29]. Within these integrated programs the individual level and organizational level are further specified into four domains. The individual level is subdivided into two domains, i.e. 1): screening and support, where identification of lifestyle related issues and support in addressing these issues is key and 2) information and education, which focuses on creating awareness about the importance of a healthy lifestyle. The organizational level also consists of two domains: 3) adjustments in the social, digital or physical environment to support a healthy lifestyle and 4) policy adjustments to facilitate and encourage a healthy lifestyle. The present study will follow this definition for an integrated approach (Fig. 2). This definition will also be used to formulate the criteria for the integrated approach.

#### Step 2. Select

The aim of the second MAP step is to discuss the lifestyle themes, derived from the focus groups and interviews in step 1, to be included in the catalogue and the criteria of the integrated approach. This will be done with an advisory board, during a group meeting. The advisory board exists of representatives of employees, employers, the Ministry of Health, Welfare and Sport, and the Ministry of Social Affairs and Employment, as well as experts from the Center for Healthy Living and National Institute for Public Health and the Environment. If necessary, themes will be renamed or reclassified, and criteria will be adjusted. With this information, the initial draft of the catalogue will be adapted.

#### Step 3. Prepare

During the preparation step the catalogue will be finalized and a cluster randomized controlled trial (C-RCT) will be prepared. In doing so, the catalogue will be pre-tested by a working group of managers and supervisors from an organization that is experienced in implementing WHP activities. This is also one of the organizations that will participate in a focus group. The working group will verify the fit of the materials to the organization and staff and they will be asked to critically review the materials and provide feedback on attractiveness, readability and understanding of the instructions. In doing so, the working group will jointly fill in a checklist. If necessary, one representative of the working group will elaborate on this completed checklist during a conversation with the researcher. Information from the checklist and conversation will be used to make changes to the materials and to finalize the catalogue. In preparation for the C-RCT, HR professionals, management, prevention workers and employees from each organization that will participate in the C-RCT will form a practice group which will review recruitment materials, promotion materials and presentations. The practice group will also support in the recruitment of workers to participate in the C-RCT by providing information and creating support among employees.

#### Step 4. Pilot

The implementation plan describes the key elements for implementation of WHP activities and describes necessary resources and relevant persons within the organization that should be involved in the implementation. A pilot-test will be conducted by the working group that also was involved in step 3, to assess the feasibility and comprehensiveness of the draft implementation plan. The working group will be asked to select one activity from the catalogue to apply the implementation plan to. The working group will go through all steps of the implementation plan as if they are implementing the activity. However, the activity will not actually be implemented. The working group will express their views on the comprehensiveness and feasibility of all elements of the implementation plan according to a checklist. A representative of the working group and a researcher will discuss the provided feedback based on the completed checklist if necessary. Information retrieved from the checklist and discussion will be used to adjust and finalize the implementation plan.

#### Step 5. Implement

Several organizations will participate in the C-RCT to evaluate the Dutch WHPP. As part of the evaluation, the program will initially only be implemented in a randomly selected half of the participating departments or locations, depending on the structure of the organization. The remaining participating departments or locations will serve as a waiting list control condition and will receive the WHPP once the trial has ended.

### Evaluation plan

For the second component of this study, the design of the effect and process evaluation will be described.

#### Study population

Dutch organizations will be recruited via the extensive network of the project team members, co-workers and branch specific networks. Inclusion criteria for participants will be: working within the participating organizations for at least 12 hours per week with a contract until the final measurement, including employees with a flexible contract or self-employed persons, who have a contract with the organization for 12 or more hours per week. Exclusion criteria will be: being on sick leave for more than 4 weeks or pregnancy.

#### Recruitment

To recruit and inform employees, different communication channels, such as intranet, newsletters, posters, videos and flyers, will be used. Workers within the participating organizations are invited for an information session, which will be either at the workplace or online. The practice group will distribute an information letter and recruitment materials among the employees approximately 4 weeks prior to the start of the C-RCT. Additionally, the practice group will distribute a link by mail or through newsletters, among their employees, so that employees can obtain more information and/or express their interest in the study to the researchers prior to the information session. Employees who expressed their interest will receive information, an eligibility checklist and informed consent by post (additional file 1). During the information sessions, researchers will explain the study purpose and design. At the end of the session, employees can ask questions to the researchers. Again, the link which employees can use to express their interest in the study will be distributed. Employees can send the signed informed consent and completed eligibility checklist prior or after the information session by post to the researchers, with a return envelope that they receive together with the informed consent. 2–4 Weeks after the information session the baseline measurement will take place for employees who are eligible and returned a signed informed consent.

#### Effect evaluation

##### Study design

The effectiveness of the Dutch WHPP will be evaluated in a two-armed C-RCT with a follow-up duration of 12 months. Clusters will be composed based on working locations or units - dependent on the organization’s structure -, to reduce contamination between the control condition and intervention condition [30]. Clusters in the intervention condition will receive the WHPP, consisting of the catalogue and implementation plan, and are asked to implement activities following the criteria of the integrated approach. Continuation of already implemented WHPPs in organizations is permitted in both the control condition and intervention condition. The Medical Ethical Committee of the VU University Medical Center (VUmc, Amsterdam, the Netherlands) approved the study protocol (2021.0402). The trial is registered in the Netherlands Trial Register (NTR) under the number NL9526. Important amendments of the protocol will be communicated to all relevant parties, i.e. the Medical Ethical Committee of the VU University Medical Center (for review and approval), participating organizations, trial registry, participants and journals. Furthermore, adverse events will be reported to the Medical Ethical Committee of the VU University Medical Center. Representatives of the department of Quality, Occupational Health and Safety, and Environment of the RIVM and/or representatives of the Ethics Committee may select this project to undergo an audit. Topics of such an audit may be the progress of the study, the planning, potential highlights and/or problems. The results of this study will be disclosed unreservedly and will be presented as articles in scientific (peer-reviewed) journals and presentations at scientific conferences.

##### Randomization and blinding

Randomization within each organization will take place at cluster level and after baseline measurements. Two independent researchers will be involved in the randomization process. The first independent researcher will assign consecutive numbers to all of the clusters within an organization. The second independent researcher will receive this list without being informed about which number corresponds with which cluster. This researcher will use a computer program to randomly assign the numbers to the intervention or control condition [31]. The first independent researcher will receive the list with numbers and their allocation to the intervention or control condition and will link this to the clusters within the organization. Then, the research team of the current study will send the program to the clusters in the intervention condition. However, the researcher involved in the data processing and analyses will be blinded for group allocation, because clusters will be re-coded by an independent researcher prior to analyses.

##### Sample size calculation

The sample size needed for the proposed study was based on finding an effect on the primary outcome, a combined lifestyle score as measured using the Simple Lifestyle Indicator Questionnaire (SLIQ) [32]. The sample size calculation was carried out including cluster correction using an estimated intra-cluster correlation coefficient (ICC) of 0.04 [33]. Based on a mean score of 7.02 (standard deviation of 1.5) on a scale of 0–10, a power of 80%, a two-sided alpha of 0.05 and an estimated number of 6 clusters per condition, 264 participants (132 per group) are needed to statistically demonstrate an effect on lifestyle of 10%. Taking into account a loss to follow-up of 20% after 12 months, a total of 330 employees (2 groups of 165) need to be included.

##### Measurements

**Fig. 3:**
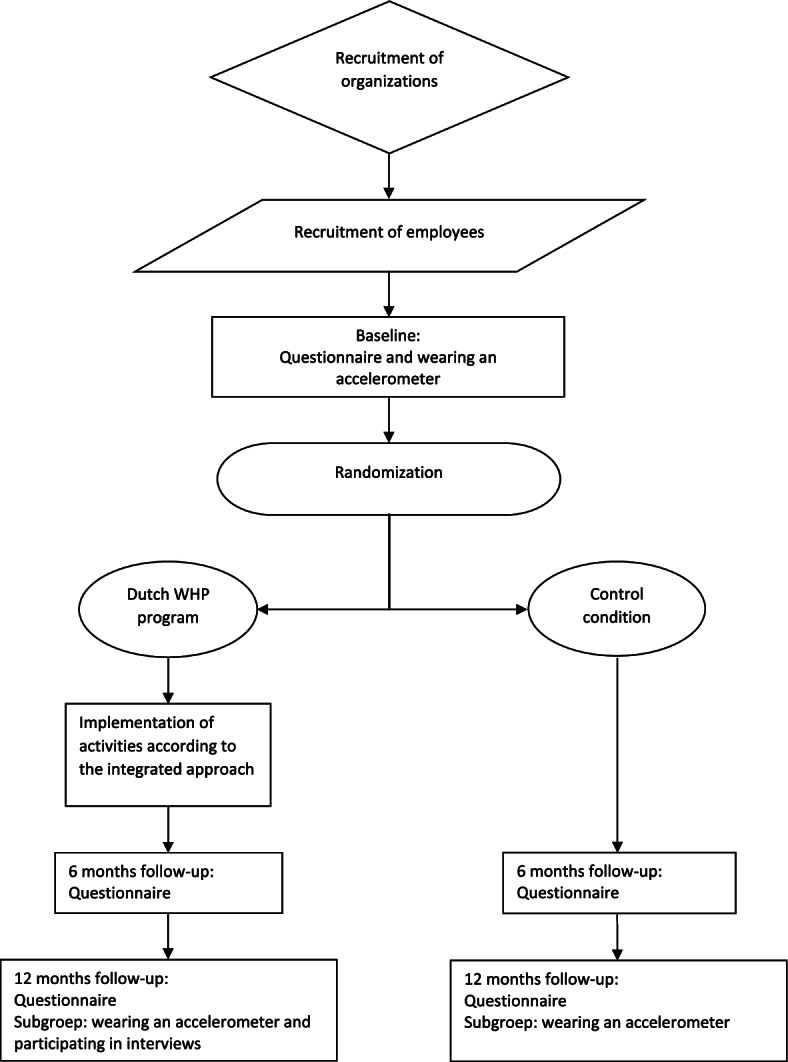
Time schedule of the C-RCT

Participants in both conditions receive online questionnaires at baseline, and at 6 and 12 months of follow-up. Additionally, a subgroup of the participants will be asked to wear a triaxial accelerometer for 7 days at baseline and 12 months (Fig. 3). The study population will include participants from various educational backgrounds. To ensure that all participants, including those with low (health) literacy, will be able to understand and complete the questionnaire, the questionnaire will be simplified. To maintain the validity of the questionnaire, the nature of the questions will not be adjusted. Words that might be difficult to read or understand will be replaced by better readable and understandable words.

##### Handling and storage of data

Data will be collected by online questionnaires and triaxial accelerometry. Data will be handled confidentially and in compliance with the General Data Protection Regulation (in Dutch: AVG). Raw anonymised data from the accelerometers will be analysed by the UKK Institute in Finland, a processing agreement is drawn up and signed for this purpose. Facilities for storage and back up of the data of the National Institute of Public Health and the Environment (Rijksinstituut voor Volksgezondheid en het Milieu) will be used. Daily backups are made. To ensure confidentiality, data will be pseudonymised. The unique pseudonym for every participant will not be based on the participant’s initials and birth date. A secured database, only accessible for the RIVM researchers involved in this study, will include the link between personal data and the specific pseudonym. At the end of the project, contact data and names of participants will be deleted form this database. Other data will be preserved for 15 years after the project ended. Due to the expected absence of (high) risks for participants of this study, the establishment of a data monitoring committee is not necessary.

###### Primary outcome measure

Lifestyle

Overall lifestyle behavior will be measured with the reliable and validated Simple Lifestyle Indicator Questionnaire (SLIQ) [32, 34]. The SLIQ provides a global lifestyle score and consists of five components: nutrition (3 questions), physical activity (3 questions), alcohol consumption (3 questions), smoking status (2 questions), and stress (1 question) [32]. The Cronbach alphas measured for nutrition and physical activity were 0,58 and 0,60 respectively [32]. As the SLIQ is only available in English it will be translated to Dutch according to the back translation method, derived from the guidelines of Guillemin et al. [35]. Two translators will independently translate the SLIQ from English to Dutch. An independent translator and one of the researchers (DS) will compose a consensus version. This Dutch translation will be back translated to English by two other translators, who are unaware of the original SLIQ. Again a consensus translation will be composed by the same independent translator and researcher. The original SLIQ and the back translated English version will then be compared and changes will be made to the Dutch SLIQ if necessary. Furthermore, cultural adaptations will be made, e.g. examples of physical activity will be adjusted if a sport is not common in the Netherlands. For each lifestyle component in the SLIQ, a score of 0–2 is assigned yielding a total score of 0–10 for the overall lifestyle score, where 0 stands for the most unhealthy lifestyle and 10 the most healthy lifestyle possible.

###### Secondary outcome measures

Secondary outcome measures include physical activity (both occupational and non-occupational), nutrition, sleep, stress, work-life balance, need for recovery, perceived general health, and well-being.

###### Physical activity

A subgroup of participants in both the intervention and control condition will be asked to wear a triaxial accelerometer (RM42 or Actigraph GT9X Link) to objectively measure physical activity at baseline and at 12 months. Participants will wear the same accelerometer at baseline and 12 months. Total minutes of both occupational and non-occupational light, moderate and vigorous activity per day will be measured as well as total minutes of occupational and non-occupational sedentary behavior, i.e. sitting and lying, and number of breaks from sitting per day. Participants will be asked to wear the accelerometer device for 24 hours on 7 consecutive days on their hip [36]. They will also keep a diary to note the date, wearing time, sleep time, working time, and time spent cycling or exercising. Raw acceleration data measured will be analyzed by using the validated mean amplitude deviation (MAD) and angle for posture estimation (APE) algorithms or the Actilife 6 Software [37–39]. Additionally, the valid and reliable Short QUestionnaire to Asses Health-enhancing physical activity (SQUASH) will be included in the questionnaire at baseline, 6 and 12 months [40]. The SQUASH questionnaire measures habitual physical activity levels during a regular week in the past month of four different physical activity domains: commuting, occupational, household and leisure time [40]. For each domain, employees will be asked to indicate the frequency (days per week), self-reported intensity (light, moderate or vigorous) and average duration (hours and minutes) of the activity per day. For each domain, activities will be subdivided into three age-dependent intensity categories (i.e., light/moderate/vigorous), corresponding to the metabolic equivalents (METs) derived from Ainsworth’s compendium of physical activities. Total minutes per week of moderate-to-vigorous physical activities will be calculated by summing the time spent on at least moderate intensity activities across the three domains of commuting, household and leisure time. Moreover one question regarding sedentary behavior will be added, to gain insight in the time spend sitting on an average day (hours and minutes).

###### Nutrition

Nutrition will be measured using six questions derived from the PIAMA Birth Cohort study [41]. One question focuses on the average amount of sugary drinks consumed per week during a regular month. The other questions involve consumption of small and large snacks, both sweet, savory and deep-fried, measuring the average amount of snacks consumed per week during a regular month.

###### Sleep

The Medical Outcomes Study Sleep scale (MOS-SS), a reliable and valid measurement instrument, will be used to assess important aspects of sleep perceived by participants [42]. In total eight aspects of sleep can be measured with the MOS-SS. For this study four aspects will be measured, i.e. sleep quantity, optimal sleep, sleep disturbance, and somnolence. Sleep quantity is scored by the average hours of sleep per night for the last 4 weeks. When a participant reports 7–8 hours of sleep, it is considered as optimal sleep, which leads to a score of 1 on this scale, more or less hours of sleep lead to a score of zero. Sleep disturbance and somnolence are scored on a 6 point scale and converted to a score between 0 and 100, in which a higher score indicates more of the concept being measured. In addition, sleep quantity, time to fall asleep and waking up during sleep will be measured using the triaxial accelerometer.

###### Stress

Stress will be measured using the stress sub-scale of the short version of the Depression Anxiety and Stress Scale (DASS-21) [43]. The stress sub-scale of the DASS-21 consists of seven statements, measuring overall stress during the past week. Responses will be summed into a scale score ranging from 0 to 21, with a higher score representing more stress. Validation of the DASS-21 has been performed in a non-clinical setting [44]. The Cronbach alpha measured for stress was 0,84 [44].

###### Work-life balance

The work-life balance will be measured by the short version of the negative work-home interference scale of the Survey Work-home Interference Nijmegen (SWING), a valid and reliable instrument with a Cronbach alpha of 0,85 [45, 46]. This scale consists of 4 items for which participants are asked to indicate how often their work-life negatively interferes with their home-life on a 4-point scale (0–3). Scores will be summed and averaged, resulting in a score between 0 and 3, in which 3 is the most negative work-home interference possible.

###### Need for recovery

Need for recovery will be measured using the corresponding subscale of the Questionnaire on the Experience and Evaluation of Work [47] The need for recovery scale is valid for the measurement of (early symptoms of) fatigue after work and a Cronbach alpha of 0,88 was measured [48]. The scale consists of 11 questions to be answered on a dichotomous scale (yes/no). The total score is standardized to a score between 0 and 100, in which 100 represents the highest need for recovery.

###### General health

Perceived general health will be measured using the subscale ‘general health perceptions’ of the RAND-36, which is a widely used and validated instrument to measure health-related quality of life [49]. The Cronbach alpha of the general health perception subscale was 0.81 [49]. General health is measured by 5 items on a 5 point scale. Answers will be coded, summed, and then transformed to a 0 to 100 scale with a higher score indicating a better health status.

###### Well-being

Well-being will be assessed by the 5-item World Health Organization Well-Being Index (WHO-5), which has shown good construct validity in various settings [50]. The questionnaire consists of five statements to be answered on a 6 point rating scale (0–5). The total score (0–25) is multiplied by 4 to achieve a scoring of 0–100 where 100 represents the best imaginable well-being.

###### Potential confounders and effect modifiers

Data on potential confounders and effect modifiers will be assessed by questionnaire including age, gender, highest educational level attained, marital status, type of work (blue/white collar), working conditions (i.e. working from home), and working days and hours per week.

##### Data analysis

First, descriptive statistics (means, standard deviations, or frequencies) at baseline will be performed for all relevant variables. The effect of the Dutch WHPP on the primary and secondary outcomes will be determined by performing longitudinal linear mixed models, adjusting for baseline differences of the outcome measure. Differences in the primary and secondary outcomes at 6 and 12 months between the WHPP condition and the control condition will be analyzed. Main analyses will be performed following the intention-to-treat principle including all available data of the participants regardless their compliance to the program.

#### Process evaluation

##### Study design

To understand the success or failure of the implementation of the integrated Dutch WHPP and its activities, a process evaluation will be conducted among the clusters in the intervention condition. Two process evaluation models will be combined, as these complement each other [51, 52]. Using the framework of Wierenga et al. (2012), recruitment, reach, dose delivered, dose received, fidelity, satisfaction, maintenance and context will be evaluated. As implementation strategy and participants’ mental models are expected to play an important role in the success or failure of the implementation, these components from the framework of Nielsen and Randall (2013) will be added to the initial framework. Data will be collected by means of mixed methods, combining quantitative and qualitative methods.

##### Measurements

A monitoring chart will be completed by the employer during the whole 12-month follow up. This monitoring chart collects information on the implemented WHP activities, time needed for preparation of implementation, the way employees were informed about the activities and in case of individual-based activities, the number of sessions and attendance of employees. At 6 months and 12 months follow up, questions regarding process outcomes will be included in a questionnaire for employees. Observations at the workplace will take place at baseline and between 10 and 12 months follow up, to observe which environmental activities were implemented and to see if employees were stimulated to participate in visible manners, i.e. posters and flyers. Additionally, interviews with employers and employees about the implementation process will be conducted between 10 and 12 months follow-up. The following process indicators will be measured:

###### Recruitment

Provides insight into the sources and procedures used to approach and stimulate employees to participate. Recruitment will be measured by observations at the workplace, a monitoring chart, interviews with employers and questionnaires among employees.

###### Reach

The proportion of employees who were aware of the integrated Dutch WHPP and the activities implemented at the workplace. Reach will be measured by means of questionnaires among employees.

###### Dose delivered

The proportion of the intended Dutch WHPP activities that is delivered by the employer to the employees. This component will be measured with the observations at the workplace and the monitoring chart.

###### Dose received

The extent to which employees were engaged in the Dutch WHPP activities. The dose received will be measured by means of the monitoring chart and questionnaires.

###### Fidelity

Compliance to the criteria of the integrated approach and compliance to the implementation plan will be measured. Information will be collected by conducting interviews with employers and the monitoring chart.

###### Satisfaction

The opinion and satisfaction about the Dutch WHPP. Employees will grade the program in the questionnaires and further information will be collected by means of interviews with employees.

###### Maintenance

The degree to which the activities and the integrated Dutch WHPP are continued within the organization. Information concerning this component will be collected by means of interviews with employers.

###### Context

Determinants of implementation which can either hinder or facilitate the implementation of the Dutch WHPP and its activities. Information on this component will be yielded by means of questionnaires and interviews with employers and employees.

###### Implementation strategy

The roles and behaviors of the key stakeholders e.g. support from management to participate in WHP activities and the perceived degree of employee involvement in the implementation of the integrated Dutch WHPP and its activities. Information will be yielded by interviews with the employers and employees and the monitoring chart.

###### Participants’ mental models

Perceptions and appraisals from the employees and employers about the integrated Dutch WHPP and its activities. It defines how employees and supervisors respond to the activities and identifies whether potential conflicting agendas may influence the behaviors and outcome of the Dutch WHPP. Information will be collected by means of interviews with employees and employers and questionnaires.

##### Data analysis

For the questionnaires, monitoring charts and systematic observations descriptive analyses will be performed and presented in mean (SD) and percentages, this includes the recruitment, reach, dose delivered, dose received, fidelity, satisfaction, context, implementation strategy and participants’ mental models. Satisfaction of the Dutch WHPP will be assessed using a rating scale of 0–10, in which 0 indicates the lowest satisfaction possible and 10 the highest satisfaction. To determine dose received we will calculate 1) the percentage of employees that had participated at least once in an individual-based activity, 2) the percentage of employees who indicated that they made use of or were exposed to an environmental activity, and 3) the percentage of employees that fulfils 1 and 2 and is therefore seen as being compliant to the integrated WHPP, i.e. they received the complete intervention. The interviews will be recorded and transcribed verbatim. Transcripts will be coded independently by two researchers by means of thematic coding. This analysis includes the constructs context, fidelity, maintenance, implementation strategy and participants’ mental models. To evaluate the context component, the Consolidated Framework for Implementation Research will be used. Analyses will be done using MAXQDA.

## Discussion

This paper describes the protocol of tailoring the Lombardy WHP Network to the Dutch context and the design of the effect and process evaluation.

The Lombardy WHP Network has shown promising results in the improvement of lifestyle behaviors of employees and has been successful in the implementation of integrated activities in order to stimulate a healthy lifestyle among their employees [18]. These results, especially regarding the successful implementation and participation, are promising, since poor reach of target groups and poor implementation are common among WHPPs and weaken the potential effect [5]. An integrated approach and the availability of a catalogue, where an employer can choose the activities that best suit the organization and its staff, are expected to be effective and successful in implementation. Therefore, a valid translation, retaining the core elements of the Lombardy WHP Network, i.e. the catalogue and the integrated approach, is important to create a successful Dutch WHPP. The MAP is a systematic approach that assists in adapting and tailoring interventions, while retaining core elements of the original intervention [22]. Multiple other interventions, often aimed at HIV prevention, have been adapted using the MAP approach and have been found effective [53, 54]. Therefore, the proposed use of the MAP is seen as a strength. It guides researchers systematically through the five stages of adaptation, which allows for sufficient documentation and a clear overview.

The bottom-up approach, where employers and employees will take part in the development of the catalogue and implementation plan that will be applied is another strength of the proposed study. This approach ensures that the adapted program suits the target population, the employers and employees. Their input will be taken into account during the different steps of the adaptation. They will provide information about relevant lifestyle-themes and potential barriers and facilitators and pretest the materials that will be used. In addition, an advisory board will be involved in several steps of the process, accounting for information and feedback from several relevant perspectives. However, the program will be specifically tailored to the organizations participating in this study. Even though we aim for participating organizations to vary in sector, we cannot guarantee wide application in other organizations and other sectors.

The chosen study design for the effectiveness evaluation, i.e. a C-RCT, is common in public health research [55, 56]. However, it comes with methodological limitations, such as risk of selection and dilution bias and participants within one cluster that tend to be more alike compared to participants in other clusters, and can therefore not be assumed to be independent [30, 57]. In this study we account for this in the design by letting recruitment take place before randomization of the clusters and in the analysis by performing longitudinal multilevel analyses according to the intention-to-treat principle [57, 58]. The study design allows for single blinding, in which the researcher involved in the analyses will be blinded for group allocation. This is a strength of the proposed study.

Overall, literature regarding the adaptation of WHPPs is scarce. Therefore, a process evaluation is valuable as it will provide insight into the success as well as failure aspects of the translation to the Dutch WHPP and its implementation [59]. Results from the process evaluation can thus be used to further improve the implementation plan, that is part of the Dutch WHPP, and to improve program outcomes [60].

The Lombardy WHP Network, an integrated approach for health promotion at the workplace is proven to be effective in the improvement of lifestyle behaviors. However, further scientific evidence about the effectiveness of an integrated approach in the occupational setting is scarce. Following the MAP approach, the good practice Lombardy WHP Network will be systematically tailored to the Dutch context, retaining its core elements. Next, effectiveness and process of implementation will be evaluated. This proposed study to the effectiveness and implementation process of the tailored integrated Dutch WHPP will contribute to filling the gap in literature and practice regarding integrated WHP approaches.

## Supplementary Information

**Additional file 1.** Participant information letter, including informed consent form.

**Additional file 2.** Completed SPIRIT checklist.

## Data Availability

Data sharing is not applicable to this article as no datasets were generated or analyzed during the current study.

## Abbreviations

NCDs: Non-communicable diseases
WHP: Workplace Health Promotion
WHPP: Workplace Health Promotion Program
MAP: Map of Adaptation Process
C-RCT: Cluster Randomized Controlled Trial
NTR: Netherlands Trial Register
SLIQ: Simple Lifestyle Indicator Questionnaire
ICC: Intra-cluster correlation coefficient
MAD: Mean amplitude deviation
APE: Angle for posture estimation
MOS-SS: Medical Outcomes Study Sleep scale
DASS-21: Depression Anxiety and Stress Scale
NSWING: Survey Work-home Interference Nijmegen
WHO-5: World Health Organization Well-Being Index
