# Supplementary material for: A study protocol of the adaptation and evaluation by means of a cluster-RCT of an integrated workplace health promotion program based on a European good practice
Source: BMC Public Health. 2022 May 21;22:1028. doi: 10.1186/s12889-022-13352-0 (PMC9123680; doi:10.1186/s12889-022-13352-0)
Supplement: Supplementary file 1 — Additional file 1. Participant information letter, including informed consent form. [file 12889_2022_13352_MOESM1_ESM.pdf]

Subject information for participation in medical research

Work towards Vitality!

Official title: Effectiviteit van integrale gezondheidsbevordering op het werk

Commented [A1]: This is the **full title of the study in Dutch**, as stated on the ABR form.

Introduction

Dear Sir/Madam,

With this letter, we would like to ask you to take part in a medical study. Participation is voluntary. You have received this letter because you have are employed by one of the participating organizations in this study.

You can read about the medical study in this information sheet, what it means for you, and what the pros and cons are. It is a lot of information. Can you please read the information and decide if you want to take part? If you want to take part, complete the form in Appendix D.

Ask your questions

You can take your decision based on the information in this information sheet. We also suggest that you do this:
- Put your questions to the investigator who gave you this information.
- Talk to your partner, family or friends about this study.
- Ask questions to the independent expert.
- Read the information on www.rijksoverheid.nl/mensenonderzoek.

1. General information

The National Institute for Public Health and Environment (RIVM) has set up this study. Below, we always call RIVM the 'sponsor'.

The Medical Ethics Review Committee of the VU University Medical Center (VUmc, Amsterdam, the Netherlands) has approved this study.

2. What is the purpose of the study?

In this study we look at whether an integrated approach for workplace health promotion leads to a better lifestyle and health of employees. A healthy lifestyle can include healthy eating, exercising, non-smoking and relaxation.

3. What is the background of the study?

An unfavorable lifestyle is common within the Netherlands. We know that an unfavorable lifestyle can lead to several diseases, such as diabetes and coronary diseases. Even during working hours, it is important to work on a healthy lifestyle, especially since many people spend a great amount of time at the workplace. Therefore we are looking if an integrated workplace health promotion program to improve lifestyle, is effective.

4. What happens during the study?

How long will the study take?
Are you taking part in the study? It will take about 12 months in total. See Appendix B for a schematic display of the study.

Step 1: are you eligible to take part?
You are eligible to participate when:
- You work for at least 12 hours per week
- You have a contract until the final measurement

You are not eligible to participate when:
- You are pregnant
- You are on sick leave for more than four weeks

Do you have a flexible contract or are you self-employed? You are eligible to participate when:
- You work for a participating organization until the final measurement
- You work for at least 12 hours a week for a participating organization.

Step 2: The intervention
For this intervention, your employer receives a catalogue with several activities to promote a healthy lifestyle among employees. Your employer will chose some of these activities to implement. This means that you can participate in such activities (for example a training). It can also mean that the physical work environment is adapted (for example the company restaurant).

Within the participating organizations, working locations or units - dependent on the organization’s structure – will be distributed in 2 groups:
- Group 1: Working locations or units within this group will receive the catalogue and will implement several activities. Employees within these working locations or units can voluntarily participate in these activities.
- Group 2: Working locations or units within this group will not receive the catalogue.

A draw determines which group your working location or unit will be in, this draw will be conducted by one of the researchers from the research team.

Step 3: study and measurements
When you decide to participate, you will receive an online questionnaire at the start of the study and after 6 and 12 months. Completing the questionnaire will take approximately 15 minutes. Furthermore, you will be asked to wear a triaxial accelerometer for seven days at the start and after 12 months. Additionally you will complete an activity diary during these seven days. With this information, the researchers can determine the effect of the integrated approach on the lifestyle and health of employees. Moreover, you may be invited for an interview after 12 months, in which we will ask how you experienced the program. These interviews will be audio-recorded. The interview will be coded and transcribed. As soon as the interview is transcribed, the recording will be deleted. We ask that you give us permission to do so.

What is different from the ordinary situation?
This study is not very different from the ordinary situation. The only difference is that the employer receives a catalogue with activities to promote a healthy lifestyle during this study. There may be a greater focus on lifestyle and occupational health and you may be able to participate in one or more activities.

5. What agreements do we make with you?

We want the study to go well. That is why we want to make the following agreements with you:

- You should contact the investigator in these situations:
  - You are hospitalised or get treatment in a hospital.
  - You suddenly have problems with your health.
  - You no longer want to take part in the study.
  - Your telephone number, address or email address changes.
  - You stop working for your current employer
  - You get pregnant

6. What side effects, adverse effects or discomforts could you experience?

You will not experience any side effects, adverse reactions, or discomfort as a result of this study.

7. What are the pros and cons if you take part in the study?

Taking part in the study can have pros and cons. We will list them below. Think about this carefully and talk to other people about it.
Possible pros:
- You can participate in activities that promote a healthy lifestyle at work
- You can improve your lifestyle. Which eventually can lead to a better health
- You will help researchers to get more insight in the effectiveness of an integrated workplace health promotion program

- You will receive a summary of the results at the end of the study

Possible cons:

- Completing the three questionnaires takes time (approximately 15 minutes for each questionnaire)
- Completing the activity diary takes time
- Wearing the triaxial accelerometer on your hip might be inconvenient

You do not wish to participate in the study?
It is up to you to decide if you wish to participate in the study. You do not wish to participate? This will have no further consequences for you. The researchers will not inform your employer or supervisor whether or not you are participating.

8. When does the study end?

The investigator will let you know if there is any new information about the study that is important to you. The investigator will then ask you if you want to continue to take part.

In these situations, the study will stop for you:

- You have completed all questionnaires and measurements, this is after 12 months.
- You want to stop participating in the study yourself. You can stop at any time. Report this to the investigator immediately. You do not have to explain why you want to stop.
- You will go on maternity leave before the end of the study.
- You stop working for your current employer.
- One of the following authorities decides that the study should stop:
    - RIVM,
    - the government, or
    - the Medical Ethics Review Committee assessing the study

What happens if you stop participating in the study?
The investigators use the data that have been collected up to the moment that you decide to stop participating in the study.

The entire study ends when all the participants have finished.

9. What happens after the study has ended?

Will you get the results of the study?
About 12 months after you took part in the study, the investigator will inform you about the most important results of the study.

10. What will be done with your data?

Are you taking part in the study? Then you also give your consent to collect, use and store your data.

What data do we store?

We store these data *<use if applicable and complete if necessary>*:

- your name
- your gender
- your mail address
- your age
- information that we collect during the study, including accelerometry data

Why do we collect, use and store your data?

We collect, use and store your data to answer the questions of this study. And to be able to publish the results.

How do we protect your privacy?

To protect your privacy, we give a code to your data. We only put this code on your data. We keep the key to the code in a safe place at the RIVM. When we process your data, we always use only that code. Even in reports and publications about the study, nobody will be able to see that it was about you.

Who can see your data?

Some people can see your name and other personal information without a code. These are people checking whether the investigators are carrying out the study properly and reliably. These persons can access your data:

- The RIVM researchers who conduct this study.
- An auditor who is hired by or works for the RIVM.
- The Healthcare and Youth Inspectorate.

These people will keep your information confidential. We ask you to give permission for this access.

Processing of data by an external organization

To process the data from the accelerometers the coded data will be shared with the UKK institute in Finland. This data cannot be traced back to you. This organization guarantees a confidential and careful treatment of the research data.

For how long do we store your data and body material?

We store your data for 15 years with the RIVM. Your contact details will be deleted as soon as the study is finished.

Can you take back your consent for the use of your data?

You can take back your consent for the use of your data at any time. This applies to the use in this study. But please note: if you take back your consent, and the investigators have already collected data for research, they are still allowed to use this information.

Do you want to know more about your privacy?

- Do you want to know more about your rights when processing personal data? Visit www.autoriteitpersoonsgegevens.nl.
- Do you have questions about your rights? Or do you have a complaint about the processing of your personal data? Please contact the person who is responsible for processing your personal data. For the present, this is:
  - RIVM, see Appendix A for contact details, and website.
- If you have any complaints about the processing of your personal data, we recommend that you first discuss them with the research team. You can also contact the Data Protection Officer of the RIVM. Or you can submit a complaint to the RIVM.

Commented [A2]: Reference can also be made to the website of the institution and the website of the sponsor

Where can you find more information about the study?

You can find more information about the study on the following website (s).

- https://www.zonmw.nl/nl/onderzoek-resultaten/preventie/programmas/project-detail/effectiviteitsonderzoek/making-workplace-health-promotion-work-translation-implementation-and-evaluation-of-an-integrated/
- https://www.trialregister.nl/trial/9526

11. Will you receive compensation if you participate in the study?

Participation in this study will not cost you anything. Neither will you get any compensation if you take part in this study. But you will receive a small gift.

12. Are you insured during the study?

You are not additionally insured for this study. Because taking part in the study has no additional risks.

13. Do you have any questions?

You can ask questions about the study to the research team. Would you like to get advice from someone who is independent from the study? Then contact [name of expert]. He/she knows a lot about the study, but is not a part of this study.
Do you have a complaint? Discuss it with the investigator. If you prefer not to do so, please submit your complaint to the RIVM. Appendix A tells you how to do this.

14. How do you give consent for the study?

You can first think carefully about this study. Then you tell the investigator if you understand the information and if you want to take part or not. If you want to take part, fill in the consent form that you can find with this information sheet. You and the investigator will both get a signed version of this consent form.

Thank you for your attention.

16. Appendices to this information

A. Contact details

B. Schedule of study interventions/description of study interventions or overview of measurements

C. Consent form

Appendix A: contact details for RIVM

Executive researcher:
Do you have any questions after reading this information letter? If so, please contact the executive researcher Denise Smit:

Contact details Denise Smit

Principal investigator:
The National Institute for Public Health and Evironment (RIVM) is responsible for this study, the principal investigator is [name]

Contact details principal investigator

Independent professional:
Would you like independent advice about participating in this study? If so, please contact an independent expert.

Contact details independent professional

Complaints:

If you have a complaint about the conduct of the study, you may submit your complaint in writing using the contact information below (please clearly state at the top of your letter or your email that it is a complaint and the subject of your complaint):

Contact details

Data Protection Officer of the institution:

For more information about your rights:
Contact details

Appendix B: Diagram of study interventions and/or overview of measurements/description of study interventions

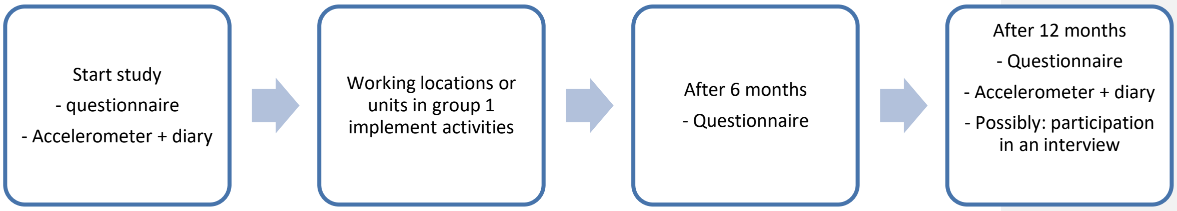

Appendix C: Informed consent form – subject

Belonging to

Work towards Vitality

- I have read the information sheet. I was able to ask questions. My questions have been answered well enough. I had enough time to decide if I wanted to take part.
- I know that taking part is voluntary. I also know that at any time I can decide not to take part in the study. Or to stop taking part. I do not have to explain why.
- I give consent to collect and use my data as mentioned in the information letter. The investigators only do this to answer the question of this study.
- I know that some people will be able to see all of my data to review the study. These people are mentioned in this information sheet. I give consent to let them see my data for this review.
- I know that the information I provide will be processed in a coded manner and that my employer will not have any insight into it.
- I give consent for the information I provide to be kept for 15 years within the RIVM. My contact details will be deleted at the end of the study.
- I give consent for people to approach me to participate in interviews for this study. I give permission for an audio recording to be made and processed during this interview. The audio recording will contain the following personal data: voice and name. The interview will be developed in coded form and the recording will be deleted once the recording is developed.
- I know that RIVM is cooperating with the UKK institute in Finland for the analysis of the accelerometers. They will only receive coded data. This organization guarantees confidential and careful handling of the research data. In Finland the privacy rules of the EC apply.

- I want to take part in this study.

My name is (subject): ……………………………….

Signature: ……………………… Date : __/__/__

<u>Contact details</u>

E-mail address:…………………………………………………………………………….

Adress:………………………………………………………………………………………

<u>Additional information</u>

Employer:……………………………………………………………………………………

Department:…………………………………………………………………………………

Location (if applicable):……………………………………………………………………

I declare that I have fully informed this subject about the study mentioned.

If any information becomes known during the study that could influence the subject's consent, I will let this subject know in good time.

Investigator name (or their representative): ....
Signature:……………………… Date: __/__/__

The study subject will receive a complete information sheet, together with a signed version of the consent form.
